# *Ab initio* molecular dynamics of liquid water using embedded-fragment second-order many-body perturbation theory towards its accurate property prediction

**DOI:** 10.1038/srep14358

**Published:** 2015-09-24

**Authors:** Soohaeng Yoo Willow, Michael A. Salim, Kwang S. Kim, So Hirata

**Affiliations:** 1 Department of Chemistry, University of Illinois at Urbana-Champaign, 600 South Mathews Avenue, Urbana, Illinois 61801, USA; 2 Center for Superfunctional Materials, Department of Chemistry, Ulsan National Institute of Science and Technology (UNIST), Ulsan 689-798, Korea; 3 CREST, Japan Science and Technology Agency, 4-1-8 Honcho, Kawaguchi, Saitama 332-0012, Japan

## Abstract

A direct, simultaneous calculation of properties of a liquid using an *ab initio* electron-correlated theory has long been unthinkable. Here we present structural, dynamical, and response properties of liquid water calculated by *ab initio* molecular dynamics using the embedded-fragment spin-component-scaled second-order many-body perturbation method with the aug-cc-pVDZ basis set. This level of theory is chosen as it accurately and inexpensively reproduces the water dimer potential energy surface from the coupled-cluster singles, doubles, and noniterative triples with the aug-cc-pVQZ basis set, which is nearly exact. The calculated radial distribution function, self-diffusion coefficient, coordinate number, and dipole moment, as well as the infrared and Raman spectra are in excellent agreement with experimental results. The shapes and widths of the OH stretching bands in the infrared and Raman spectra and their isotropic-anisotropic Raman noncoincidence, which reflect the diverse local hydrogen-bond environment, are also reproduced computationally. The simulation also reveals intriguing dynamic features of the environment, which are difficult to probe experimentally, such as a surprisingly large fluctuation in the coordination number and the detailed mechanism by which the hydrogen donating water molecules move across the first and second shells, thereby causing this fluctuation.

Nearly all previous atomistic simulations of liquids have been performed with molecular mechanics (MM) force fields^1^,^2^,^3^,^4^,^5^,^6^,^7^,^8^,^9^ or with forces supplied by on-the-fly density-functional theory (DFT) calculations^10^,^11^,^12^,^13^ or by quantum mechanical/molecular mechanical (QM/MM) calculations^14^. MM is limited to systems that are already well characterized, since force parameterization requires fitting to such *a priori* information. DFT is a great improvement over MM because the former can be directly applied to any liquid systems. Yet, many of DFT functionals lack intermolecular dispersion interactions, despite their importance as a cohesive force in many liquids, or otherwise do not have sufficiently high accuracy. In fact, DFT simulations of bulk water have predicted a considerably elevated melting temperature (*T*_m_ ≈ 400 K)^15^ and thus the existence of its liquid phase in rather unrealistic conditions^12^. Although *ad hoc* dispersion corrections have been shown to improve the description of liquid water^16^,^17^, such improvements are generally nonsystematic, and in these cases DFT falls short of being a predictive theory. One is thus interested in applying systematically improvable theories, such as *ab initio* many-body perturbation (MP) and coupled-cluster (CC) theories, to the simulation of nature’s most important liquid, i.e., water, with the eventual goal of reproducing all its observed structural, dynamical, electronic, and spectroscopic features simultaneously, accurately, and from first principles.

An application of *ab initio* MP or CC theory to a bulk liquid has not been possible because of its prohibitive cost. The situation has changed with the advent of the so-called embedded-fragment method^18^, which enables a systematic, fast, and parallel-executable application of an *ab initio* theory to large molecular clusters, crystals, and even liquids^19^,^20^,^21^. The method divides a weakly interacting system into overlapping molecular dimers (“fragments”), which are embedded in the electrostatic environment of the whole system, and then applies well-developed molecular theories and software to these fragments to reconstruct an array of whole-system properties, with a computational cost scalable with respect to both system and computer sizes.

Fujita *et al.*^19^ reported the first *ab initio* Born–Oppenheimer molecular dynamics (BOMD) simulation of liquid water with the embedded-fragment scheme known as the fragment-molecular-orbital (FMO) method^22^. They employed Hartree–Fock (HF) theory and the 6-31G* basis set as the *ab initio* theory, which does not describe electron correlation and thus lacks the dispersion interaction. The effect of three-body interactions was assessed by Komeiji *et al.*^23^ in a water cluster of (H_2_O)_32_ and that of electron correlation by Mochizuki *et al.*^24^ using second-order many-body perturbation (MP2) theory and the 6-31G* basis set on (H_2_O)_64_. Brorsen *et al.*^20^,^21^ repeated FMO-BOMD calculations of liquid water using more rigorous formulas for atomic forces at the HF and DFT level. All of these studies considered only the radial distribution functions and, owing to the smallness of the basis set employed, it is doubtful that the calculated results have much quantitative value. Perhaps the largest-scale electron-correlated calculation of liquid water performed thus far is an isobaric-isothermal Monte Carlo (MC) simulation at the MP2 level using a mixed Gaussian-planewave basis set^25^. Under the ambient conditions, it predicted a reasonable density of 1.02 g/cm^3^, but did not provide any other meaningful experimental comparison such as detailed structures, diffusion rate, vibrational spectra, or dynamics of water molecule.

In the present work, we perform BOMD simulations of liquid water using on-the-fly atomic forces obtained with thus-far the most accurate electronic structure theory for such simulations, i.e., the *ab initio* spin-component-scaled MP2 (SCS-MP2) method^26^ with the aug-cc-pVDZ basis set. The necessary speedup of the *ab initio* calculation is achieved by the embedded-fragment method known as the binary-interaction method (BIM)^27^ specifically extended for condensed-phase simulations. The choice of the theoretical level is based on our observation that it accurately reproduces the water-water interaction potentials of the coupled-cluster singles, doubles, and noniterative triples [CCSD(T)] with the aug-cc-pVQZ basis set, which is nearly exact. This theory is, therefore, expected to achieve an equally accurate quantum electronic description of the structural, dynamical, spectroscopic, and electronic properties of liquid water.

We apply this method to the radial distribution function, self-diffusion coefficient, monomer structure and its variation, coordination number and its fluctuation, intershell exchange mechanism, dipole moment and its distribution, as well as infrared (IR) and Raman spectra of liquid water (neat liquid H_2_O) under periodic boundary condition. We show that the simulations at the temperature (*T*) of 250 K and the density (*ρ*) of 1 g/cm^3^ predict the aforementioned properties in good agreement with those observed at ambient conditions. The results explain the shapes and widths of the OH stretching bands in the IR and Raman spectra, including the difference between the isotropic and anisotropic Raman components, which is known to reflect the different local hydrogen-bond environments surrounding the OH oscillators. They furthermore reveal intriguing dynamical features of the system, which are difficult to probe experimentally, such as a large fluctuation of the coordination number and the mechanism and time frame in which water molecules move across the first to second shell and thereby cause this fluctuation. They also inform us about the dipole moment and its distribution in the liquid phase as well as its breakdown into the permanent and induced components.

The properties calculated in this work range from the structural to dynamical to spectroscopic to electronic properties, which have hardly been studied simultaneously and with reasonable accuracy by any previously existing simulation methods. Therefore, the present work can provide a useful benchmark against which some controversial issues on collective properties of liquid water can be reexamined.

## Results

### Potential energy surface

**Figure 1:**
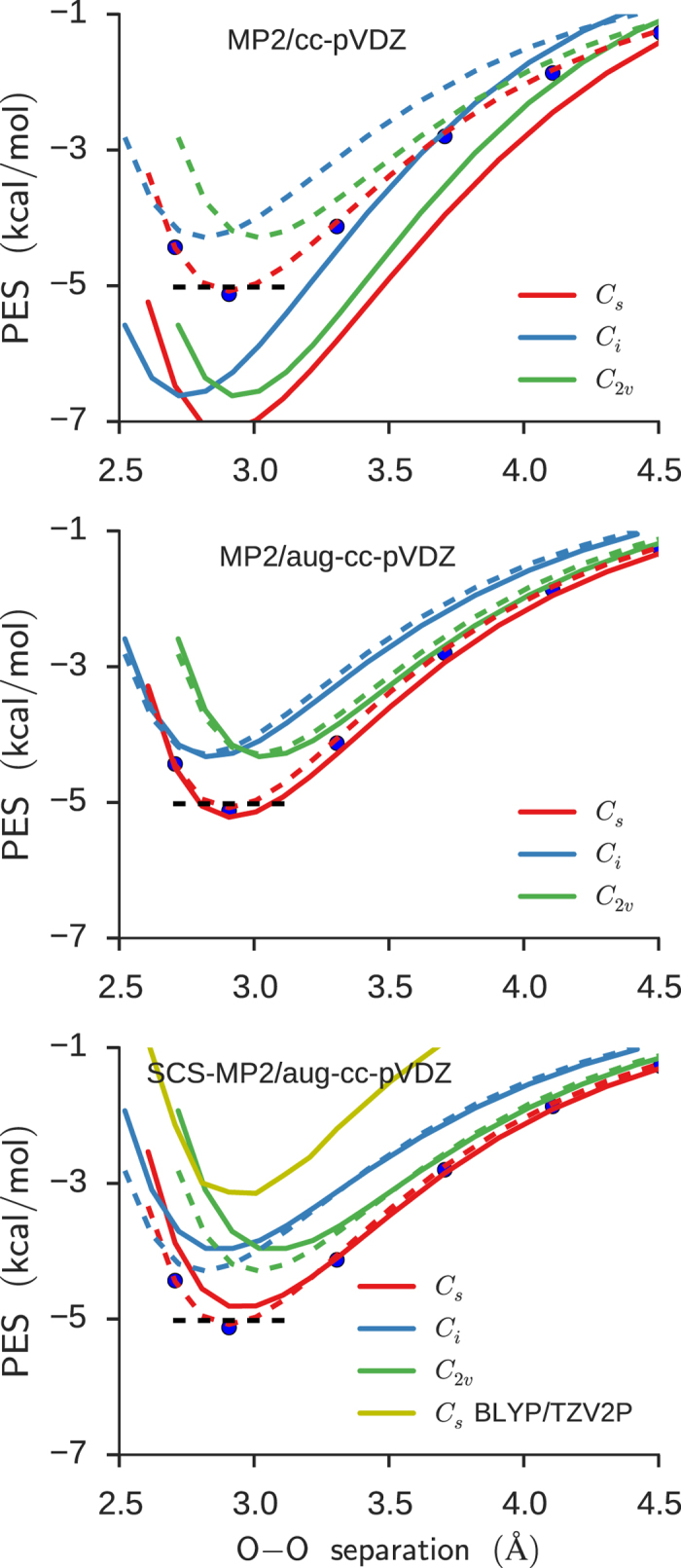
Potential energy surfaces (PES’s). The PES’s of water dimers in the *C*_*s*_, *C*_*i*_, and *C*_2*v*_ geometries as a function of the oxygen-oxygen separation (*R*_OO_) were obtained at various theoretical levels (solid curves). The dimer geometries were generated by varying *R*_OO_, with all other geometrical parameters frozen at their MP2/aug-cc-pVTZ optimized values under the appropriate symmetry constraint. The accurate results from MP2/aug-cc-pVQZ (dashed curves) and CCSD(T)/aug-cc-pVQZ (dots) are superimposed. The binding energy at CCSD(T)/CBS is indicated as dashed black lines.

The fidelity of BOMD simulations for bulk systems largely hinges on the reliability of the potential energy surface (PES) obtained from the electronic structure method, and hence verifying the latter for intermolecular interactions is a crucial prerequisite for such simulations. Figure 1 plots the PES’s of the water dimer in three geometrical configurations (having *C*_*s*_, *C*_*i*_, or *C*_2*v*_ symmetry) as a function of the O–O separation (*R*_OO_). The binding energy (Δ*E*) and equilibrium O–O separation (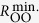) of the optimized *C*_*s*_ structure are listed in Supplementary Table S1. Compared with the accurate reference data of MP2/aug-cc-pVQZ or CCSD(T)/aug-cc-pVQZ, the MP2/cc-pVDZ (top panel of Fig. 1) overestimates the intermolecular attractions by as much as 50%, whereas the PES’s of MP2/aug-cc-pVDZ (middle panel) and SCS-MP2/aug-cc-pVDZ (bottom panel) are reasonable. This underscores the importance of diffuse basis functions in describing intermolecular forces; namely, basis sets without diffuse basis functions such as 6-31G* are inadequate for BOMD simulations of liquid water and water clusters^19^,^24^. On the other hand, DFT calculations with the Becke–Lee–Yang–Parr (BLYP) functional and diffuse basis sets tend to underestimate Δ*E* considerably and are also inadequate (bottom panel and Supplementary Table S1).

SCS-MP2/aug-cc-pVDZ (bottom panel) describes the attractive tail of the PES slightly better than MP2/aug-cc-pVDZ. Its repulsive parts are a little too strong, making the PES’s somewhat shallower and equilibrium *R*_OO_ longer than the reference data. Nonetheless, the value of Δ*E* predicted by SCS-MP2/aug-cc-pVDZ (4.86 kcal/mol) is closer to the complete-basis-set (CBS) limit of CCSD(T) of 5.02 kcal/mol (dashed lines) than the value obtained at MP2/aug-cc-pVDZ (5.26 kcal/mol), while the MP2-calculated Δ*E* seems to converge more accurately at the correct limit with a further basis-set extension^28^,^29^,^30^.

Note that the calculated values of Δ*E* and 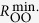 in Supplementary Table S1 do not include the zero-point vibrational energy (ZPVE) corrections or nuclear quantum corrections, which have the effect of decreasing Δ*E* and increasing 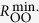 by 0.032 Å^31^. Since such corrections are not at present considered in our BOMD simulations, we find SCS-MP2/aug-cc-pVDZ convenient because its Δ*E* (4.86 kcal/mol) is smaller and its 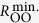 (2.942 Å) is longer than those of CCSD(T)/CBS (5.02 kcal/mol and 2.910 Å) and thus expected to be closer to experimental values that include the ZPVE effect^32^,^33^. Needless to say, the use of a large basis set such as aug-cc-pVQZ in *ab initio* electron-correlated BOMD simulations takes a prohibitive computational cost and is out of the question.

In this article, therefore, we primarily used SCS-MP2/aug-cc-pVDZ to generate atomic forces. We additionally used MP2/cc-pVDZ as the secondary method to assess the effect of overestimated Δ*E* and underestimated 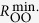 on the simulation results.

### Radial distribution function

No *a priori* information exists about the phase diagram of water for the *ab initio* potentials. The first step is, therefore, to identify where the liquid phase boundaries exist. One approach is to determine the melting temperature computationally^15^,^16^. Another is to vary the temperature such that the BOMD simulation reproduces the experimental liquid structure at ambient conditions^12^. The liquid structure is inferred from the radial distribution function (RDF), which can be observed by neutron or X-ray scattering experiment^34^,^35^ and serves as a fingerprint of local structures and thus phases. We first performed BOMD simulations at *T* = 300 K, which produced a gas-phase-like RDF, and then reduced the temperature until the calculated RDF coincided with that of the observed result for the liquid phase at ambient conditions.

**Figure 2:**
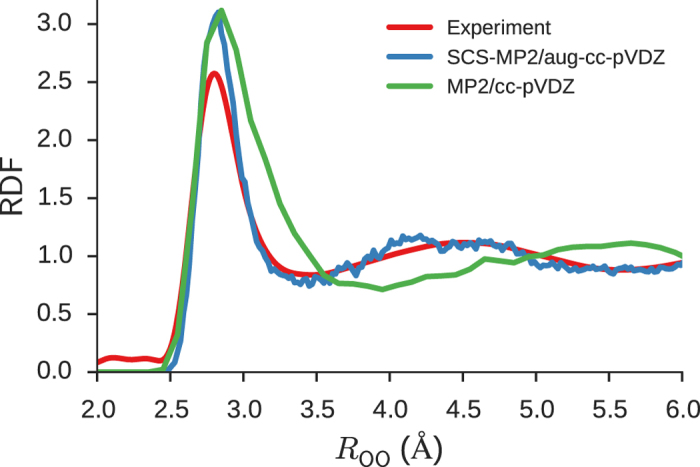
Oxygen-oxygen radial distribution function. The oxygen-oxygen radial distribution functions (RDF’s) of liquid water were obtained from the BOMD simulations at *T* = 250 K and *ρ* = 1 g/cm^3^ and were compared with the experimental result^34^ at *T* = 25 °C.

Figure 2 shows the oxygen-oxygen RDF obtained from the SCS-MP2/aug-cc-pVDZ simulation at *T* = 250 K. It reproduces the experimental RDF of the liquid phase observed at *T* = 25 °C obtained from the X-ray diffraction technique^34^. The calculated position of the first peak is close to the experimental value (~2.8 Å) and the agreement of RDF in the region of *R*_OO_ > 3.2 Å is striking. The slight difference in the calculated peak intensity from the experimental one at *R*_OO_ = 2.8 Å can be diminished by taking into account the nuclear quantum effect, as noted from the previous path-integral molecular dynamics simulation^13^. The average coordination numbers of 4.7 (*R*_OO_ ≤ 3.4 Å) or 4.4 (*R*_OO_ ≤ 3.3 Å) are in excellent agreement with the experimental values^34^. We therefore conclude that the SCS-MP2/aug-cc-pVDZ simulation at *T* = 250 K and *ρ* = 1 g/cm^3^ provides an accurate description of the structural features of liquid water at ambient conditions.

In contrast, the RDF of MP2/cc-pVDZ at *T* = 250 K exhibits an excessively broad first peak, which is a manifestation of an over-coordinated local structure with an average coordination number of as large as 8 (*R*_OO_ < 3.7 Å). This, in turn, is due to too strong an attractive intermolecular interaction predicted by MP2/cc-pVDZ, as evidenced by Fig. 1. This first peak is followed by the second peak, which is further displaced at longer *R*_OO_, indicating that the second shell is pushed outward by the overcrowded first shell. Hence, the MP2/cc-pVDZ description of liquid water structure is nonphysical and this is largely due to the smallness of the basis set.

The isobaric-isothermal MC simulations at the MP2 level with a mixed Gaussian-planewave basis set was performed by Del Ben *et al.*^25^. They yielded the liquid density (*ρ* = 1.02 g/cm^3^) slightly larger than the correct value. This is in qualitative agreement with our simulation, in which the average pressure is found to be −0.6 ± 0.5 GPa at *T* = 250 K and *ρ* = 1.0 g/cm^3^; the larger calculated liquid density at 1 bar means that a negative pressure is required to maintain it at 1 g/cm^3^. The RDF calculated at *T* = 295 K by Del Ben *et al.* was, however, overstructured, suggesting that the liquid is super-cooled, whereas the RDF obtained from our simulation is much closer to experiment. The source of the quantitative difference is hard to pinpoint because there are many approximations involved in both studies. We speculate, however, that the most likely primary source is the different choices of the basis set, in particular, the use of a pseudopotential in the preceding HF calculation in Del Ben *et al.*

### Self-diffusion coefficient

Whereas the RDF characterizes time-averaged local structures, the self-diffusion coefficient (*D*) measures dynamical properties of liquids. Its value is related to the mean square displacement (MSD) by Einstein’s diffusion equation. The calculated value of *D* ≈ 0.27 Å^2^/ps from our BOMD simulation at *T* = 250 K compares favorably with the experimental value of 0.23 Å^2^/ps at *T* = 25 °C^36^. Hence, we argue that the present simulation properly describes the dynamical properties of liquid water at ambient conditions also.

### Water molecular geometry in the liquid phase

**Figure 3:**
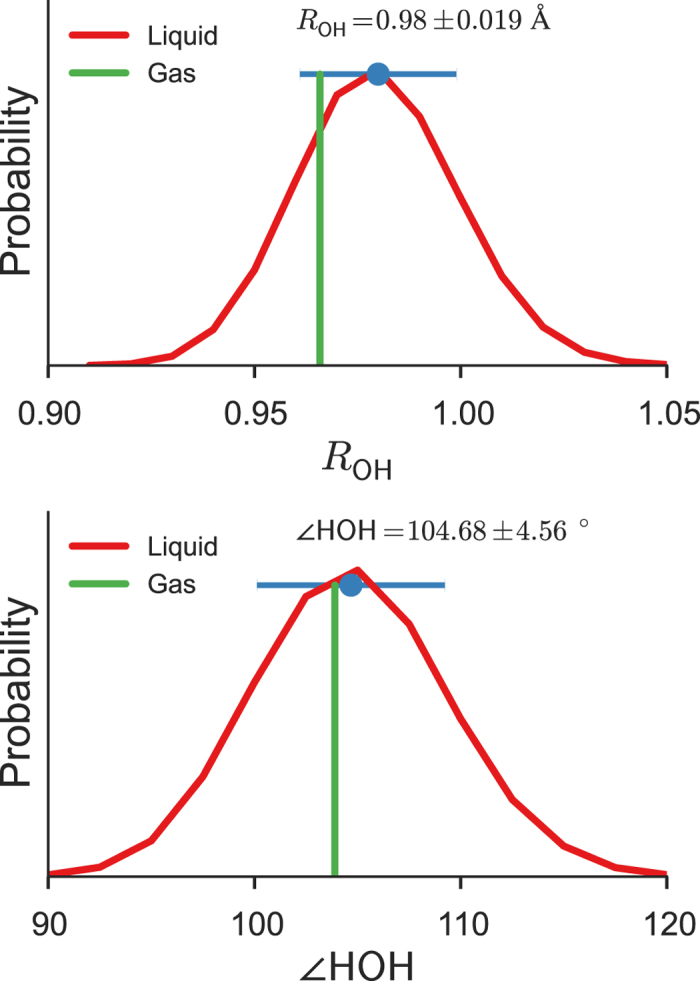
Bond lengths and angles. Distributions of bond lengths (top) and angles (bottom) of water molecules in the liquid phase, compared to the optimized geometry of the water molecule in the gas phase. The blue dots and bars indicate the averages and standard deviations.

Each water molecule undergoes a significant geometry change on going from the gas to liquid phase. The water molecule in the gas phase has the OH bond length (*R*_OH_) of 0.966 Å and HOH angle (∠HOH) of 103.9° at the SCS-MP2/aug-cc-pVDZ level, which are in good agreement with the experimental values^37^ of 0.957 Å and 104.5°. In the liquid phase, the calculated bond lengths and angles form distributions (Fig. 3) with *R*_OH_ = 0.980 ± 0.019 Å and ∠HOH = 104.7 ± 4.6°. Two experimental investigations of liquid water yielded measurements of (*R*_OH_ = 0.970 ± 0.005 Å; ∠HOH = 106.1 ± 0.9°)^38^and (*R*_OH_ = 0.983 ± 0.008 Å; ∠HOH = 104.1 ± 1.9°)^39^. Remarkably, the BOMD simulation predicts an average OH bond elongation (relative to the gas phase) of 0.014 Å, which is in excellent agreement with the measured elongation of 0.013 Å^38^. It is notable that the bond angle in liquid water ranges from 90° to 120° with a large standard deviation of 4.6°.

### Coordination numbers and intershell exchange

**Figure 4:**
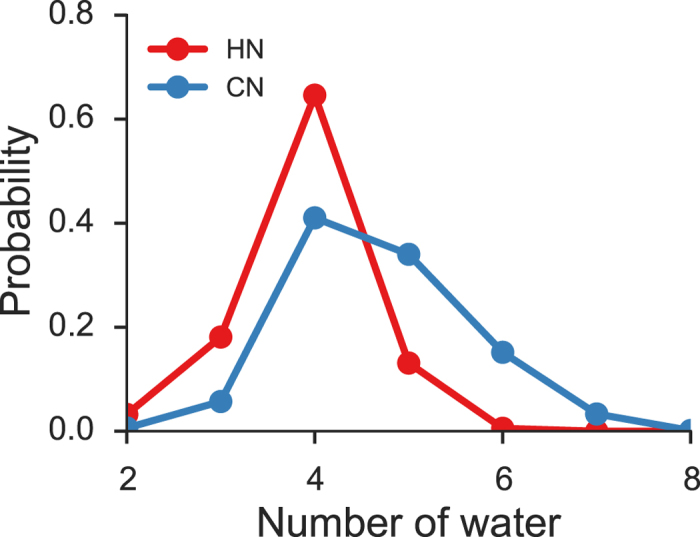
Coordination number and hydrogen-bond number. The distribution of the number (CN) of water molecules in the first shell (*R*_OO_ ≤ 3.36 Å) excluding the central molecule and the distribution of the number (HN) of hydrogen bonds with the central molecule (with a hydrogen bond being considered to exist between two water molecules when *R*_OO_ ≤ 3.36 Å and ∠HOO ≤ 40°).

**Figure 5:**
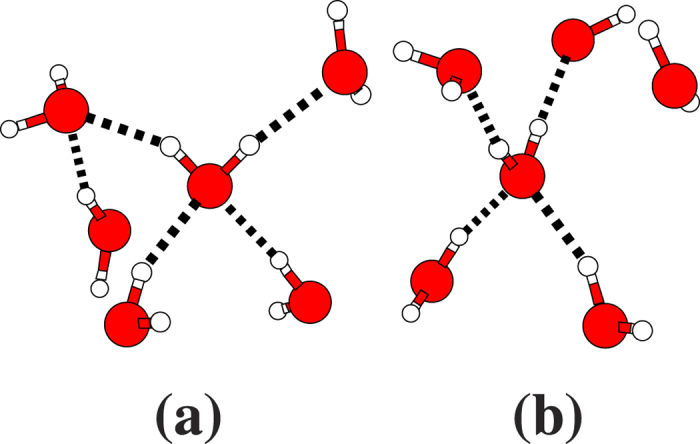
Snapshots of local structures in liquid water. The snapshots of the penta-coordinated water molecule are drawn. It is, on average, hydrogen-bonded to two H-acceptors and two H-donors. The fifth water molecule in the first shell stays non-hydrogen-bonded or weakly hydrogen-bonded. It can be (**a**) near the **D** zone or **(b)** near the **A** zone in the contour maps in Fig. 7.

Figure 4 plots the distribution of the coordination number, that is, the number of water molecules in the first shell, as well as the distribution of the hydrogen-bond number. According to our simulation, a water molecule has an average coordination number of 4.7 and an average hydrogen-bond number of 3.8. The former value is in good agreement with experiment^34^. In most cases (65%), each molecule is hydrogen-bonded to four neighbors with two serving as a H-donor (hydrogen donating water) and two as a H-acceptor (hydrogen accepting water), while the remaining neighbors in the first shell stay non-hydrogen-bonded or weakly hydrogen-bonded, as shown in Fig. 5.

The coordination number displays a surprisingly large fluctuation (4.7 ± 0.9), ranging from mono-coordination to octa-coordination. Overall, tetra-coordination is the most common (41%), followed by penta-coordination (34%) and hexa-coordination (15%). Tri-coordination (6%) and hepta-coordination (3%) are also significant, while bi-coordination (0.6%), octa-coordination (0.1%), and mono-coordination (0.01%) occur much less frequently. A majority of the penta- and higher-order coordinations have one or more non-hydrogen-bonded or weakly hydrogen-bonded water molecules in the first shell, judging from the rapid falloff of the hydrogen-bond number after four.

**Figure 6:**
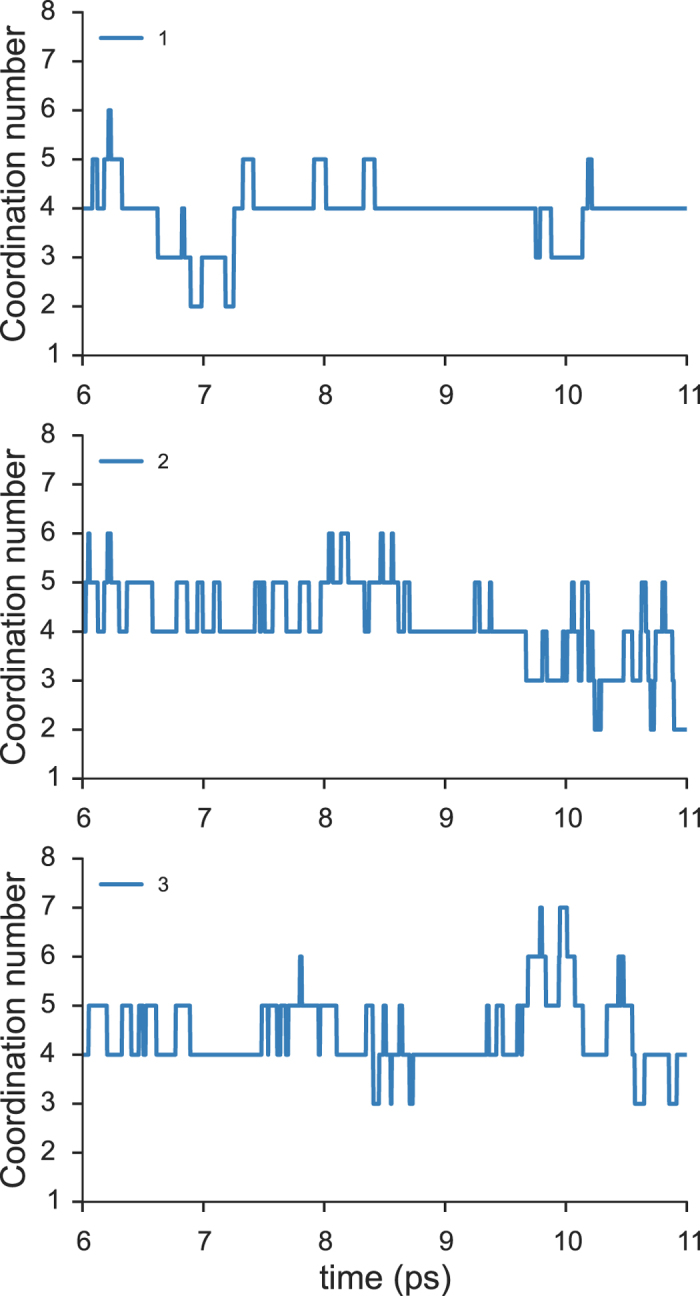
Fluctuation of the coordination number. The number of water molecules in the first shell (*R*_OO_ ≤ 3.36 Å) as a function of time is tracked down for three selected water molecules.

Figure 6 illustrates the time-evolution of the coordination numbers of three randomly selected water molecules. The large and rapid variation in each case may be related to the significant density fluctuations observed even in a nm-sized droplet of liquid water, as discussed in a recent study^40^.

**Figure 7:**
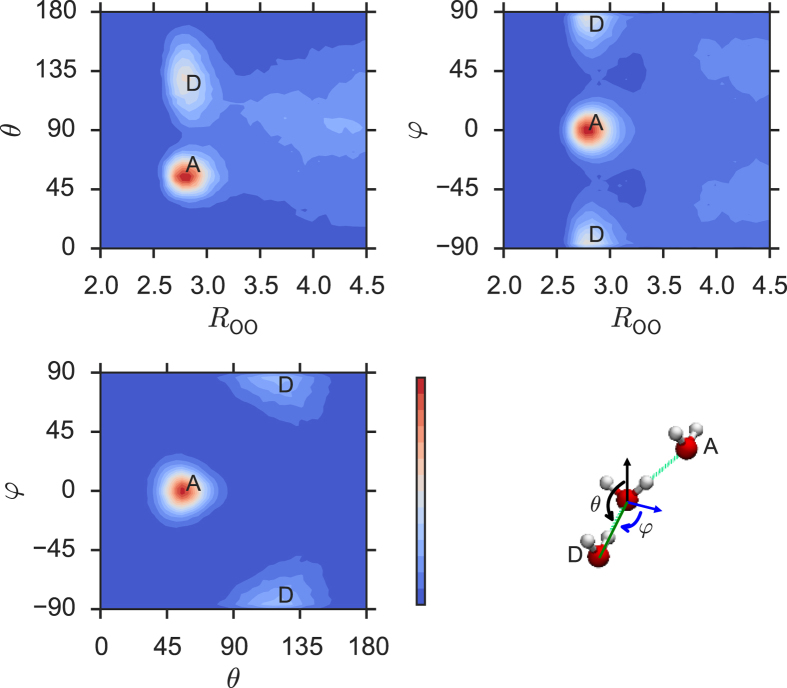
Oxygen-atom population. The (*R*_OO_, *θ*) and (*R*_OO_, *φ*) contour maps of the oxygen atom population within the second-shell RDF peak (*R*_OO_ ≤ 4.5 Å) and the (*θ*, *φ*) contour map within the first shell (*R*_OO_ ≤ 3.4 Å). *R*_OO_, *θ*, and *φ* are spherical coordinates as defined above. **D** and **A** stand for the hydrogen-donating (H-donor) and accepting (H-acceptor) water molecules, respectively.

Figure 7 shows the (*R*_OO_, *θ*) and (*R*_OO_, *φ*) population contour maps of the oxygen atoms for *R*_OO_ up to the second-shell RDF peak (*R*_OO_ ≤ 4.5 Å) as well as the (*θ*, *φ*) population contour map for the first shell (*R*_OO_ ≤ 3.4 Å). They reveal that H-acceptors (the **A** zone) are found around *R*_OO_ = 2.8 Å, *θ* = 50°, and *φ* = 0°, while H-donors (the **D** zone) populate around *R*_OO_ = 2.8 Å, *θ* = 120°, and *φ* = ±90°. A significant population density is observed at *θ* = 100° around *R*_OO_ = 3.4 Å in the (*R*_OO_, *θ*) contour. This is the likely path through which a non-hydrogen-bonded or weakly hydrogen-bonded neighbor in the **D** zone migrates from the first shell to the second shell and vice versa, causing the fluctuation in the coordination number.

Our BOMD trajectory data such as those shown in Fig. 6 provide even more detailed information about key events responsible for the coordination number fluctuation. Let us define a 5-4-5 hetero-exchange as the process by which a penta-coordinated water molecule loses its non-hydrogen-bonded neighbor to become tetra-coordinated, and a *different* water molecule takes its place (hetero-exchange), restoring the penta-coordinated state. This event is frequently observed in our trajectories, lasting an average of *ca.* 100 fs and occurring to a penta-coordinated species approximately every 1.0 ps. Similarly, we use the term the 4-5-4 self-exchange to describe the transient attachment of a non-hydrogen-bonded water molecule to the first shell of a tetra-coordinated water, followed by the detachment of the same molecule. This process occurs, on average, approximately every 1.5 ps with a duration of *ca.* 80 fs. The 5-4-5 self-exchange and 5-6-5 hetero-exchange are also noted at an average frequency of every few ps. The high frequency and long duration of these exchange events involving tetra- and penta-coordinated species are due to their greater stability and thus longer average lifetime.

### Dipole moments

The dipole moment of a water molecule in the liquid phase has been under debate for a long time, partly because there is no experimental means of measuring it directly. Accurate theoretical predictions are, therefore, extremely valuable, but previously reported computed values ranged widely from 2.6 to 3.0 D^11^,^41^,^42^. An earlier DFT-MD study placed it at 2.66 D^10^, but later revised it to 2.95 D^41^. The latter, larger predicted value may be traced to the significantly overestimated polarizability characteristic of DFT. Subsequently, Badyal *et al.*^43^ determined the partial charge on the hydrogen atom as *q*_H_ = 0.5 ± 0.1 a.u. by X-ray diffraction. On this basis, they suggested the dipole moment of 2.9 ± 0.6 D, which had such a large error bar that it encompassed nearly all computationally suggested values.

**Figure 8:**
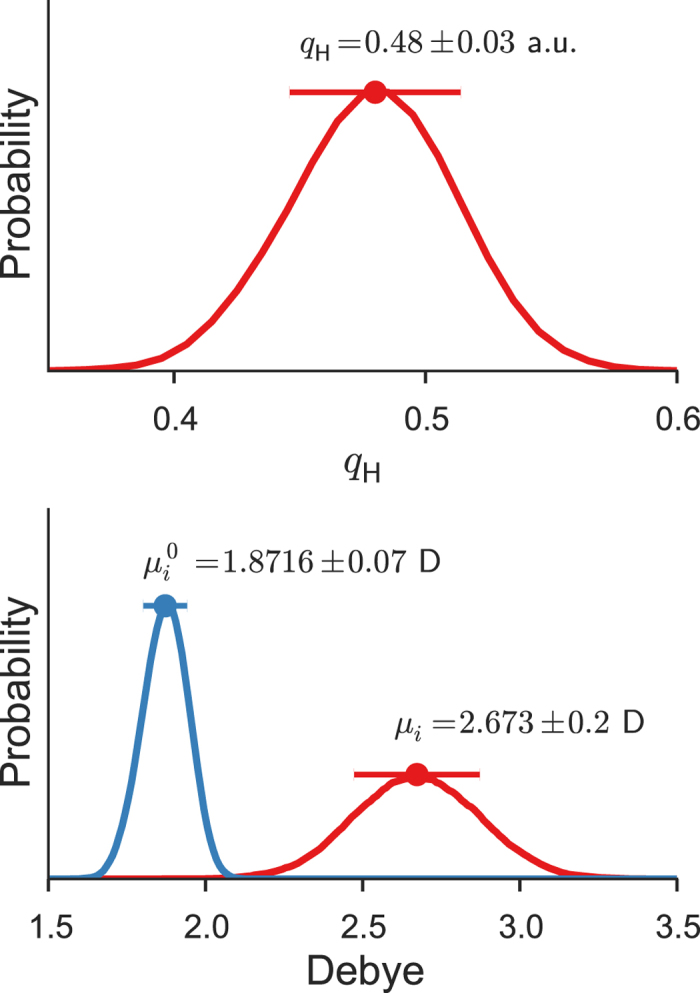
Dipole moments and hydrogen partial charge. The distribution of the partial charges of the hydrogen atoms (top) and the distribution of the permanent (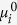) and total (*μ*_*i*_) dipole moments (bottom) of the water molecules in the liquid phase. The total dipole moment is obtained with the embedding field within the cut-off distance *R*_EF_ = 9 Å, whereas the permanent dipole moment with the field turned off.

The dipole moment of the water molecule in the gas phase calculated by SCS-MP2/aug-cc-pVDZ is 1.878 D, which agrees well with the experimental value of 1.855 D^44^. Figure 8 plots the distributions of the permanent dipole moments 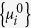 (obtained with the embedding field turned off) and the total dipole moments {*μ*_*i*_} (calculated with the embedding field within 9 Å and thus including the induced dipole moments) in the liquid phase. The permanent dipole moment 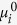 at SCS-MP2/aug-cc-pVDZ is 1.872 ± 0.07 D, whereas the total dipole moment *μ*_*i*_ is 2.67 ± 0.2 D. Hence, the induced dipole moment is significant and amounts to as much as 0.80 D on average. it could further increase slightly by the nuclear quantum effect^13^, but perhaps by not more than 0.05 D.

Furthermore, the total dipole moment is broadly distributed, showing large fluctuations from 1.8 to 3.5 D, with about 5% exceeding 3.0 D, owing more to the diversity of electrostatic fields than to the monomer geometry fluctuation. The partial charge of the hydrogen atom, *q*_H_ = 0.48 ± 0.03 a.u. (Fig. 8), is in good agreement with the mean of the experimental value of Badyal *et al.*, *q*_H_ = 0.5 ± 0.1 a.u.^43^. The difference in the total dipole moment between our study and Badyal *et al.* may be traced to the difference in the water structure: the OH bond length proposed^45^ by one of the authors of ref. 43 is 1.045 Å and is much greater than our predicted (0.980 Å) or experimental values (0.970–0.983 Å)^38^,^39^. Our predicted dipole moment (*μ*_*i*_ = 2.67 D) is in excellent agreement with other independent experimental expectation (~2.6 D)^46^ and theoretical calculation (~2.65 D)^42^. In short, the dipole moment suggested by Badyal *et al.* (2.9 D) is, in view of this and other accurate theoretical calculations, questionable and likely overestimated.

### IR and Raman spectra

The SCS-MP2/aug-cc-pVDZ BOMD simulation predicts the vibrational frequencies of the water molecule in the gas phase at 1676, 3800, and 3900 cm^−1^. Though BOMD simulations sample the fully anharmonic PES, owing to the classical treatment of vibrations, these frequencies are too high relative to the experimental values (1595, 3657, and 3756 cm^−1^)^37^,^47^, for reasons that are well understood^48^. To correct such deviations rigorously, one should use path-integral molecular dynamics^8^,^49^,^50^, which can take into account the ZPVE or quantum nuclear effects. A more empirical correction is to simply multiply the calculated frequencies by a scale factor (0.96 for the gas phase and 0.93 for the liquid phase) that can bring the calculated peak positions in better agreement with those observed experimentally.

**Figure 9:**
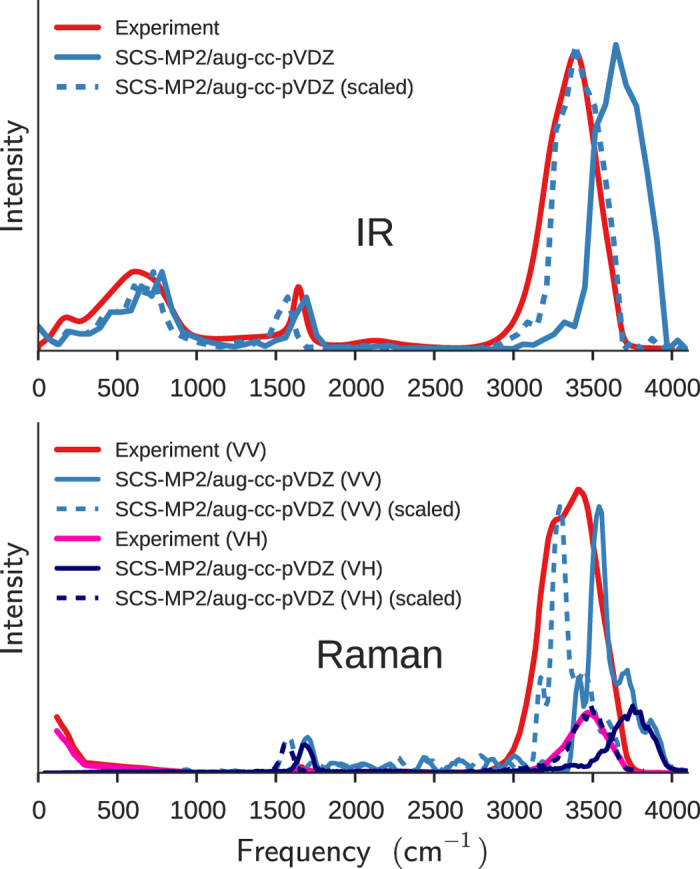
IR and Raman spectra. The IR and Raman spectra of liquid water were calculated with the SCS-MP2/aug-cc-pVDZ simulation at *T* = 250 K and *ρ* = 1 g/cm^3^. The experimental IR spectrum at *T* = 25 °C is taken from ref. 52 and the experimental VV and VH Raman spectra at *T* = 23 °C from ref. 51. The dashed curves are obtained by scaling the frequencies of the calculated spectra by 0.93 to empirically take into account the ZPVE (see the text).

The IR and Raman spectra of liquid water computed from the SCS-MP2/aug-cc-pVDZ BOMD simulation at *T* = 250 K are compared with the corresponding experimental results at ambient conditions^51^,^52^,^53^ in Fig. 9. Unlike most calculated spectra, each band in our simulated spectra is not convoluted with an assumed band shape, but is instead associated with a physically meaningful width that reflects the diversity of local environments in which the vibrations occur.

The BOMD simulation reproduces all three major features of the observed IR spectrum (top panel): the broad, intense OH stretching band above 3000 cm^−1^, the narrower peak due to the bending mode at around 1600 cm^−1^, and the manifold of intermolecular vibrations below 1000 cm^−1^.

The OH stretching band in the observed IR spectrum peaks at about 3400 cm^−1^ with a weak shoulder around 3250 cm^−1^ and a full width at half maximum (FWHM) of 375 cm^−1^ 52,53. Remarkably, the BOMD simulation predicts the OH stretching band with the correct band width (FWHM of about 375 cm^−1^) and slightly asymmetric band shape, which also agrees with the observed shape, though the calculated peak positions of 3550 and 3700 cm^−1^ are blue-shifted. It is striking that the classical “continuum” picture^48^, in which diverse instantaneous hydrogen-bond environments surrounding the OH stretching vibrations give rise to the band width, is borne out by our simulation. Our time-domain computation of the spectra should also naturally account for the motional narrowing of this band, which is estimated to be as much as 30% of the width^54^.

The OH stretching band of the observed VV Raman spectrum has bimodal peaks at about 3250 and 3400 cm^−1^ and a FWHM of about 425 cm^−1^, while the observed VH spectrum shows the OH stretching band around 3460 cm^−1^ with an asymmetric line shape and a FWHM of about 300 cm^−1^ 51. The calculated VV and VH Raman spectra are also in agreement with the observed spectra. It explains the asymmetric line shape of the OH stretching band in the VH spectrum and the double-peaked nature of the band in the VV spectrum, including the difference in peak positions between the VH and VV components, known as the Raman noncoincidence effect^55^. With the aforementioned frequency scaling by a factor of 0.93, the simulated VV and VH spectra are again in excellent agreement with their experimental counterparts both in peak positions and shapes.

It could be emphasized that our spectral simulation does not involve any adjustable parameter (which is not to say that there is no approximation) in the anharmonic force field, mode-mode coupling, polarizability tensor, etc., unlike previous analyses on the same bands^48^. Note that polarizable model potentials of water^8^,^56^ currently fall short of simultaneously accounting for polarization effects on intermolecular interactions and optical response, such as the Raman effect. Our *ab initio* method can achieve just that, although the peak positions are systematically overestimated because of the classical treatment of vibrations.

## Discussion

We have performed *ab initio* BOMD simulations of liquid water at the SCS-MP2 and MP2 levels of theory and computationally reproduced the structural properties (the radial distribution function, monomer geometry and its fluctuation) and structure-driven collective properties (the self-diffusion coefficient, coordination number and its variation, intershell exchange) as well as the electronic and response properties (permanent and induced dipole moments, IR spectra, and isotropic and anisotropic Raman spectra) simultaneously, accurately, and from first principles. The level of theory has been chosen to reproduce the CCSD(T)/aug-cc-pVQZ results of the water dimer, allowing us to study the electronic details of liquid water with unprecedented accuracy.

The calculated properties of liquid water are in good agreement with the experimental results, when the latter are available. The simulated IR and Raman spectra correctly predict the band shapes and widths of the OH stretching bands and the difference between their isotropic and anisotropic Raman components, which are exceedingly difficult with classical MD with parameterized force fields, even if they are polarizable.

For properties that are difficult to probe experimentally, our simulations provide unique insights. The average bond length and angle of the water molecule in the liquid phase are found to increase only minimally, but range from 0.92 to 1.05 Å and from 90° to 120°. The coordination number fluctuates even more greatly from mono-coordination to octa-coordination, though the tetra- (41%) and peta-coordinations (34%) are most common. Such large fluctuation in the coordination number might lead to significant density fluctuations. We have identified 5-4-5 hetero-exchange and 4-5-4 self/hetero-exchange as the key events, occurring approximately every 1–2 ps with an average duration of *ca.* 0.1 ps and causing the large fluctuation in the coordination number. Such exchanges take place as a non-hydrogen-bonded water molecule in the **D** zone migrates between the first and second shells. The total dipole moment (*μ*_*i*_ = 2.67 ± 0.2 D) of the water molecule in the liquid phase also shows a large fluctuation, ranging from 1.8 to 3.5 D, reflecting more the diversity of the electrostatic field surrounding the water molecule than the geometry fluctuation.

Hence, the present method allows us to assess the validity of uncertain results or conclusions about a whole range of liquid water properties. It can also be exploited to study other unknown collective properties of liquid water and various solvents as well as colligative properties of solute-solvents generally.

## Methods

### Born-Oppenheimer molecular dynamics

BOMD simulations were carried out in the canonical (*NVT*) ensemble with the velocity Verlet algorithm in conjunction with the Nosé–Hoover chain method^57^,^58^. A time step of 1 fs was used. An initial liquid structure of (H_2_O)_32_ in a cubic cell of side 9.858 Å was obtained from an equilibration run with the TTM3-F force field^8^ at *T* = 300 K. Using the same force field, we verified that the 32-water simulation yielded the calculated RDF which was unchanged from the result of a 512-water simulation and was, therefore, adequate. Using SCS-MP2/aug-cc-pVDZ or MP2/cc-pVDZ energies and atomic forces at *T* = 250 K, a 6-ps equilibration run was performed, after which the trajectory was sampled every 4 time steps during a 5-ps production run.

### Self-diffusion coefficient

The self-diffusion coefficient (*D*) is related to the MSD by Einstein’s diffusion equation:


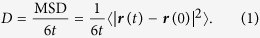


In turn, MSD was obtained by the calculation of the squared relative displacement of the oxygen atoms as a function of time *t* averaged over all water molecules in the unit cell.

### IR and Raman spectra

The IR and Raman spectra were calculated by Fourier transformation of the time-correlation functions of the system’s dipole moment and polarizability, respectively. For a direct comparison with the experimental spectra, the harmonic quantum correction was applied to the classically computed spectra^59^. IR intensity *I*_IR_(*ω*) at frequency *ω* then becomes^56^


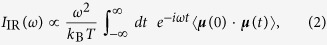


where ***μ***(*t*) is the dipole moment of the system at time *t*, defined as the sum of the dipole moments of monomers, {***μ***_*i*_}, in the unit cell,


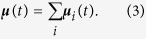


Here, the dipole moment of the *i*th monomer embedded in the electrostatic field, ***μ***_*i*_, is directly computed by the SCS-MP2 method.

The harmonic-quantum-corrected, Bose–Einstein-reduced isotropic and anisotropic Raman spectra were calculated by^56^


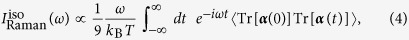



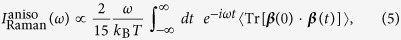


where ***α*** is the polarizability tensor of the unit cell and ***β*** = ***α*** − Tr[***α***]***I***/3 with ***I*** being the identity tensor. Each element of ***α*** is the sum of the corresponding elements of the embedded monomer polarizability tensors, {***α***_*i*_}. The latter is evaluated as the ratio of the induced dipole moment 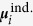 to the applied electric field ***E***, e.g.,


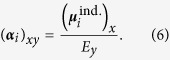


Induced dipole moments are, in turn, evaluated directly by the SCS-MP2 method for an embedded monomer with additional point charges of ∓1 a.u. placed at ***r***_cm_ ± 100 bohr along each Cartesian axis. Here, ***r***_cm_ denotes the center of mass of the monomer. The parallel-polarized (VV) and perpendicular-polarized (VH) Raman spectra, with each letter denoting the vertical (V) or horizontal (H) polarization direction of the incident and scattered light, are related to the isotropic and anisotropic components by


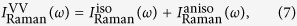



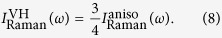


Using the classical MD with the TTM3-F force field^8^, we verified that the simulation with a 1-fs time step yields the same IR spectra as the simulation with a 0.2-fs time step. The only exception was the positions of the OH stretching bands (if not their widths and shape), in which a time-step error of up to 50 cm^−1^ (or ~1.5%) was observed.

### Energy and gradient evaluation

The potential energy, atomic gradients, and pressure of liquid water in each time step were calculated by the massively parallel implementation of the BIM method^27^ using the SCS-MP2/aug-cc-pVDZ or MP2/cc-pVDZ method.

The formalisms of BIM for condensed-phase simulations are documented in the Supplementary Information. Briefly, it divides the liquid into monomers and overlapping dimers embedded in the electrostatic environment of the liquid. The potential energies and gradients of each of the monomers or dimers, treated quantum mechanically (QM), are evaluated with SCS-MP2 or MP2 implemented in a modified nwchem^60^. They are used to reconstruct the potential energy, atomic forces, and pressure of the whole liquid under the periodic boundary condition according to Eqs (S1), (S5), and (S6) in the Supplementary Information. The embedding field (EF) consists of atomic point charges that approximate the electrostatic potential of each monomer and are self-consistently determined at the HF level. The calculation thus includes two-body electrostatic, exchange, and dispersion interactions at the SCS-MP2 or MP2 level and three-body and all higher many-body electrostatic interactions at the HF level. The truncation radii of the QM, EF, and long-range (LR) regions, as defined by Eqs (S1)–(S4), are as follows: *R*_QM_ = 7.5 Å, *R*_EF_ = 9 Å, and *R*_L*R*_ = 12 Å.

## Additional Information

**How to cite this article**: Willow, S. Y. *et al.* *Ab initio* molecular dynamics of liquid water using embedded-fragment second-order many-body perturbation theory towards its accurate property prediction. *Sci. Rep.* **5**, 14358; doi: 10.1038/srep14358 (2015).

## Supplementary Material

Supplementary Information
