# Supplementary material for: Ab initio molecular dynamics of liquid water using embedded-fragment second-order many-body perturbation theory towards its accurate property prediction
Source: Sci Rep. 2015 Sep 24;5:14358. doi: 10.1038/srep14358 (PMC4585828; doi:10.1038/srep14358)
Supplement: Supplementary Information [file srep14358-s1.pdf]

Supplementary information: *Ab initio* molecular dynamics of liquid water using embedded-fragment second-order many-body perturbation theory towards its accurate property prediction

Soohaeng Yoo Willow,[1,2,3] Michael A. Salim,[1] Kwang S. Kim*,[2] and So Hirata†[1,3]

[1]*Department of Chemistry, University of Illinois at Urbana-Champaign, 600 South Mathews Avenue, Urbana, Illinois 61801, USA*

[2]*Center for Superfunctional Materials, Department of Chemistry, Ulsan National Institute of Science and Technology (UNIST), Ulsan 680-798, Korea*

[3]*CREST, Japan Science and Technology Agency, 4-1-8 Honcho, Kawaguchi, Saitama 332-0012, Japan*

* Corresponding author: Kwang S. Kim, kimks@unist.ac.kr
† Corresponding author: So Hirata, sohirata@illinois.edu

Embedded-fragment method for condensed-phase systems

We use an implementation of the embedded-fragment methods, which have enabled a fast *ab initio* calculation of clusters[S1–S9], crystals[S10–S22], and even liquids[S23–S25] (see also Gao[S26]). The implementation is the binary-interaction method (BIM)[S12,S22].

In this method, the expression of the potential energy per unit cell, E, is given by

$$\begin{aligned} E &= \sum_{i} E_{i:Q_i} \\ &+ \sum_{i>j}^{R_{ij}\leq R_{\mathrm{QM}}} \left(E_{ij:Q_i\cup Q_j} - E_{i:Q_i\cup Q_j\cup j} - E_{j:Q_i\cup Q_j\cup i} \right) \\ &+ E_{\mathrm{C}} + E_{\mathrm{LR}}. \end{aligned} \tag{S1}$$

Here, the summations run over monomers i in the primary unit cell and j in all unit cells. $E_{i:Q_i}$ is the total energy of the ith monomer embedded in the field of point charges (Q_i). These charges are placed on all atoms of the molecules whose centers of mass fall within the distance of R_{EF} from the center of mass of the ith monomer.

$E_{i:Q_i}$ is obtained as the eigenvalue solution of the Schrödinger equation with the following Hamiltonian in some approximation (such as MP2):

$$\begin{aligned} H = &-\sum_{n} \frac{1}{2}\nabla_n^2 - \sum_{n}\sum_{A\in i} \frac{Z_A}{R_{nA}} - \sum_{n}\sum_{A\in Q_i} \frac{q_A}{R_{nA}} \\ &+ \sum_{m>n} \frac{1}{R_{mn}} + \sum_{A>B\in i} \frac{Z_A Z_B}{R_{AB}} + \sum_{A\in i}\sum_{B\in Q_i} \frac{Z_A q_B}{R_{AB}}, \end{aligned} \tag{S2}$$

where i and Q_i indicate the QM (fragment) and EF (embedding field) regions, respectively, m and n run over all electrons, A and B over atoms, R_{nA}, etc. are distances between the particles specified by the subscripts, Z_A is the Ath atomic number, and q_A is the partial charge of the Ath atomic site in the embedding field.

$E_{ij:Q_i\cup Q_j}$ is the total energy of the dimer consisting of the ith and jth monomers in the union of their two embedding fields. $E_{i:Q_i\cup Q_j\cup j}$ is the total energy of the ith monomer in the union of the embedding fields of the ith and jth monomers plus point charges on the atoms of the jth monomer. We introduce the cutoff distance $R_{ij} \leq R_{\mathrm{QM}}$ for the dimer calculations, where R_{ij} is the distance between the centers of mass of the ith and jth monomers. R_{QM} is assumed to be less than the unit-cell length, which is reasonable for simulation of liquids where the unit-cell length is large. This assumption can easily be lifted for periodic solid simulations. See Supplementary Figure S1 for the schematic drawing of these three fragments.

The first term in the right-hand side of Eq. (S1) double-counts the Coulomb interactions between the ith monomer and the atomic charges in the embedding field of the jth monomer and vice versa. These spurious interactions are eliminated by the second term only in the range of $R_{ij} \leq R_{\mathrm{QM}}$. The remaining error is removed by E_{C}, which is given by

$$E_{\mathrm{C}} = -\sum_{i>j}\sum_{A\in i}\sum_{B\in j}\frac{q_A q_B}{R_{AB}}, \quad \text{if} \quad R_{\mathrm{QM}} < R_{ij} \leq R_{\mathrm{EF}}, \tag{S3}$$

where A and B run over atoms in the ith and jth monomers, respectively. E_{LR} contains the long-range Coulomb interactions truncated at distance R_{LR}:

$$E_{\mathrm{LR}} = \sum_{i>j}\sum_{A\in i}\sum_{B\in j}\frac{q_A q_B}{R_{AB}}, \quad \text{if} \quad R_{\mathrm{EF}} < R_{ij} \leq R_{\mathrm{LR}}. \tag{S4}$$

The gradient with respect to the x-coordinate of the kth atom, x_k, can be determined by

$$\begin{aligned}\frac{\partial E}{\partial x_k} &= \sum_i \frac{\partial E_{i:Q_i}}{\partial x_k} \\ &+ \sum_{i>j}\left(\frac{\partial E_{ij:Q_i\cup Q_j}}{\partial x_k} - \frac{\partial E_{i:Q_i\cup Q_j\cup j}}{\partial x_k} - \frac{\partial E_{j:Q_i\cup Q_j\cup i}}{\partial x_k}\right) \\ &+ \frac{\partial E_{\mathrm{C}}}{\partial x_k} + \frac{\partial E_{\mathrm{LR}}}{\partial x_k},\end{aligned} \tag{S5}$$

where $\partial E_{i:Q_i}/\partial x_k$, $\partial E_{ij:Q_i\cup Q_j}/\partial x_k$, etc. are derivatives of the monomer and dimer energies, taking into account both the variations in the atomic coordinates in the QM region and embedding fields. They are readily obtained by combining the analytical-gradient and analytical-electric-field computation capabilities available in most molecular software. Using the atomic gradients, the instantaneous pressure along the x-axis at temperature T is evaluated as

$$P_x = -\frac{1}{V}\sum_k x_k \frac{\partial E}{\partial x_k} + \frac{N k_{\mathrm{B}} T}{V}, \tag{S6}$$

where N is the number of molecules in the unit cell[S27]. The validity of these energy and gradient formulas and programs was confirmed by the energy conservation in a BOMD simulation with the microcanonical ensemble.

Accuracy of the BIM

In the application of the BIM to liquid water, *ab initio* calculations are performed for the monomers and dimers of the water molecule. **Table** S1 summarizes the performance of various *ab initio* and DFT methods for the water dimer in the C_s structure, which is discussed in the

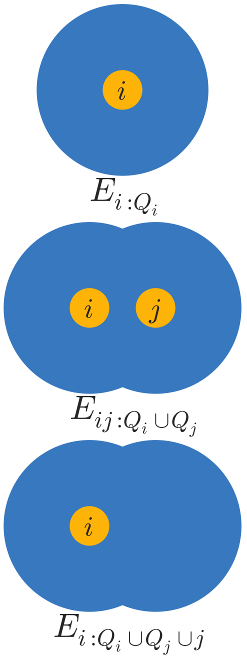

Supplementary Figure S1 | **Schematic drawing of three different fragments.** Each yellow circle represents a monomer fragment treated quantum mechanically, as embedded in the spherical embedding field (blue) consisting of atomic point charges.

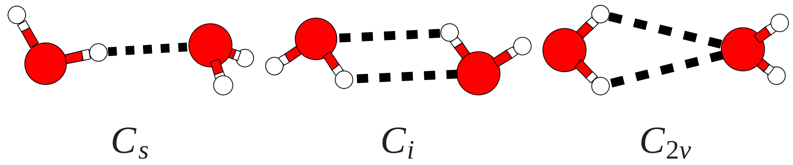

Supplementary Figure S2 | **Water dimers.** Three different water dimer structures in the C_s, C_i, and C_{2v} symmetry groups that are used for the calculations of their potential energy surfaces.

main text. **Supplementrary Figure** S2 depicts the three water dimer structures considered in the main text.

As a test of the BIM, the potential energy curve of $(H_2O)_3Cl^-$ is calculated as a function of the $Cl^- \cdots (H_2O)_3$ distance (see **Supplementary Figure** S3) at the MP2/aug-cc-pVDZ level. The results show that the BIM can also reproduce the potential energy curve of the brute-force MP2 calculation extremely accurately, even when one of the fragments is charged and strongly polarizable.

Supplementary Table S1 | **The binding energy and oxygen-oxygen distance of the water dimer.** The binding energy (ΔE in kcal/mol) and equilibrium O−O separation ($R_{OO}^{min.}$ in Å) of the optimized C_s water dimer. The frozen core approximation was employed in the (SCS-)MP2 binding energies.

Method	ΔE	$R_{OO}^{min.}$
MP2/aug-cc-pVDZ	5.26	2.917
MP2/aug-cc-pVTZ	5.18	2.907
MP2/aug-cc-pVQZ	5.09	2.900
MP2/cc-pVDZ	7.47	2.909
MP2/cc-pVTZ	6.08	2.907
MP2/cc-pVQZ	5.49	2.902
MP2/6-31G	8.38	2.869
MP2/6-31G*	7.32	2.916
SCS-MP2/aug-cc-pVDZ	4.86	2.942
SCS-MP2/aug-cc-pVTZ	4.76	2.931
SCS-MP2/aug-cc-pVQZ	4.66	2.930
BLYP/aug-cc-pVDZ	4.30	2.936
BLYP/aug-cc-pVTZ	4.17	2.943
BLYP/aug-cc-pVQZ	4.19	2.941
BLYP/TZV2P	4.75	2.966
CCSD(T)/CBS[a]	5.02	2.910

[a] Jurečka et al.[S28].

Effect of the MD time step on the IR spectra and RDF

We examined whether and to what extent the 1-fs time step alters the MD results in comparison with the 0.2-fs time step, using the TTM3-F force field[S29], which reproduces infrared (IR) spectra of liquid water. As shown in **Supplementary Figure** S4, classical MD simulations with dt = 1 fs reproduces the radial distribution function (RDF) and IR spectra nearly exactly obtained with dt = 0.2 fs. The time-step errors in the latter are on the order of ~ 50 cm^{-1} or ~ 1.5 % observed only for the frequencies of the OH stretching bands at ~ 3500 cm^{-1}, but negligible for lower-

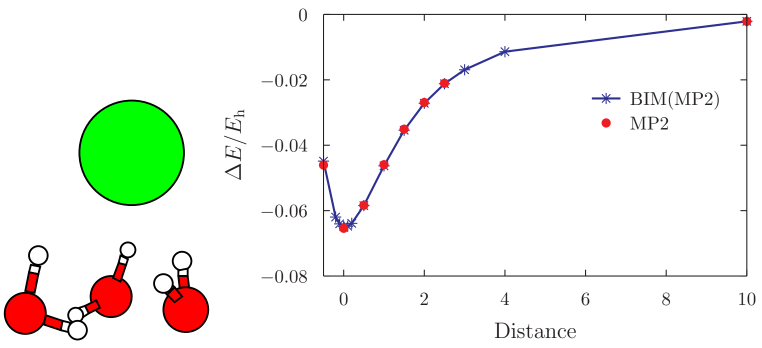

Supplementary Figure S3 | **Potential energy curve of $(H_2O)_3Cl^-$.** The structure of $(H_2O)_3Cl^-$ optimized at MP2/aug-cc-pVDZ and the potential energy curve as a function of the distance (in Å) of Cl^- from the water trimer.

frequency bands or for the shapes and widths of any band. It should be remembered that the classical treatment of the hydrogen atoms causes large errors on the order of $\sim$ 300 cm^{-1} in the frequencies of the OH stretching bands because of the nuclear quantum effects, which make the aforementioned time-step errors insignificant.

The experimental frequencies are often scaled even for a single water molecule to empirically correct anharmonicity and other effects. When such frequency scaling is applied (**Supplementary Figure** S4), the calculated and observed spectra match excellently and the differences in the OH stretching frequencies between the 0.2- and 1-fs simulations become practically negligible.

Effect of the system size on the pressure

We performed the MD simulations with the TTM-3F force field at T = 300 K and ρ = 1 g/cm^3 to quantify the effect of the system size on the pressure. A small unit cell consisted of 32 water molecules, while a large unit cell had 512 water molecules. **Supplementary Figure** S5 shows that the average pressure of the MD simulation with the small unit cell is −0.03 ± 0.54 GPa, while it is +0.02 ± 0.13 GPa with the large unit cell. The two pressures agree with each other well within the statistical uncertainty of the 512-molecule calculation. Hence, the simulation with 32 molecules per cell can reasonably describe the pressure and is expected to reproduce other properties of liquid water accurately as well.

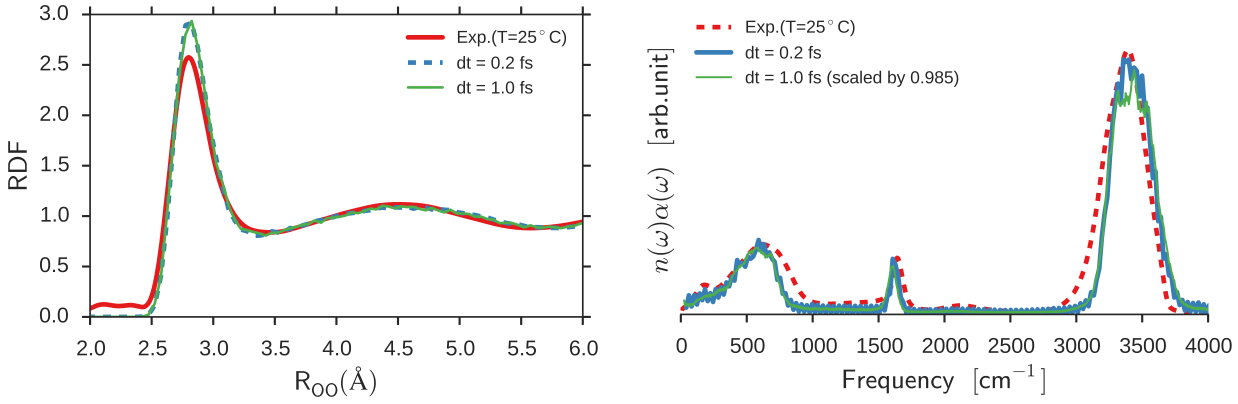

Supplementary Figure S4 | **Effect of a time step on RDF and IR spectra.** The radial distribution function (RDF) and infrared (IR) spectra of liquid water obtained from classical MD simulations with the TTM3-F force field at two different time steps.

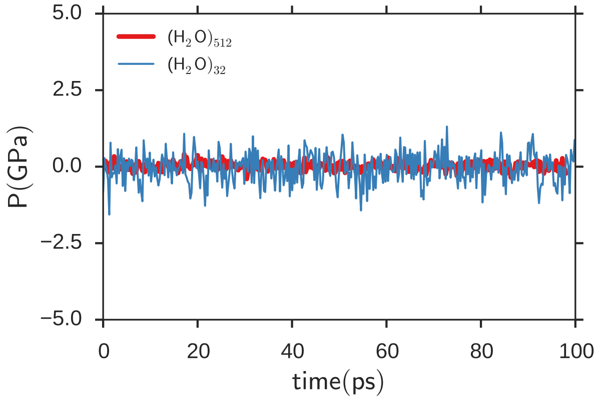

Supplementary Figure S5 | **Effect of the system size on the pressure.** The pressure of liquid water obtained from classical MD simulations with the TTM3-F force field with two different unit-cell sizes.

[S1] Kitaura, K., Ikeo, E., Asada, T., Nakano, T. & Uebayasi, M. Fragment molecular orbital method: an approximate computational method for large molecules. *Chem. Phys. Lett.* **313**, 701–706 (1999).

[S2] Zhang, D. W. & Zhang, J. Z. H. Molecular fractionation with conjugate caps for full quantum mechanical calculation of protein-molecule interaction energy. *J. Chem. Phys.* **119**, 3599–3605 (2003).

[S3] Babu, K. & Gadre, S. R. Ab initio quality one-electron properties of large molecules: Development and testing of molecular tailoring approach. *J. Comput. Chem.* **24**, 484–495 (2003).

[S4] Deev, V. & Collins, M. A. Approximate ab initio energies by systematic molecular fragmentation. *J. Chem. Phys.* **122**, 154102 (2005).

[S5] Hirata, S. *et al.* Fast electron correlation methods for molecular clusters in the ground and excited states. *Mol. Phys.* **103**, 2255–2265 (2005).

[S6] Li, W., Li, S. H. & Jiang, Y. S. Generalized energy-based fragmentation approach for computing the ground-state energies and properties of large molecules. *J. Phys. Chem. A* **111**, 2193–2199 (2007).

[S7] Dahlke, E. E. & Truhlar, D. G. Electrostatically embedded many-body correlation energy, with applications to the calculation of accurate second-order Møller–Plesset perturbation theory energies for large water clusters. *J. Chem. Theory Comput.* **3**, 1342–1348 (2007).

[S8] Richard, R. M. & Herbert, J. M. A generalized many-body expansion and a unified view of fragment-based methods in electronic structure theory. *J. Chem. Phys.* **137**, 064113 (2012).

[S9] Gordon, M. S., Fedorov, D. G., Pruitt, S. R. & Slipchenko, L. V. Fragmentation methods: A route to accurate calculations on large systems. *Chem. Rev.* **112**, 632–672 (2012).

[S10] Manby, F. R., Alfè, D. & Gillan, M. J. Extension of molecular electronic structure methods to the solid state: Computation of the cohesive energy of lithium hydride. *Phys. Chem. Chem. Phys.* **8**, 5178–5180 (2006).

[S11] Ringer, A. L. & Sherrill, C. D. First principles computation of lattice energies of organic solids: The benzene crystal. *Chem. Eur. J.* **14**, 2542–2547 (2008).

[S12] Hirata, S. Fast electron-correlation methods for molecular crystals: An application to the α, β_1, and β_2 modifications of solid formic acid. *J. Chem. Phys.* **129**, 204104 (2008).

[S13] Podeszwa, R., Rice, B. M. & Szalewicz, K. Predicting structure of molecular crystals from first principles. *Phys. Rev. Lett.* **101**, 115503 (2008).

[S14] Schwerdtfeger, P., Assadollahzadeh, B. & Hermann, A. Convergence of the Møller–Plesset perturbation series for the fcc lattices of neon and argon. *Phys. Rev. B* **82**, 205111 (2010).

[S15] Beran, G. J. O. & Nanda, K. Predicting organic crystal lattice energies with chemical accuracy. *J. Phys. Chem. Lett.* **1**, 3480–3487 (2010).

[S16] Collins, M. A. Ab initio lattice dynamics of nonconducting crystals by systematic fragmentation. *J. Chem. Phys.* **134**, 164110 (2011).

[S17] He, X., Sode, O., Xantheas, S. S. & Hirata, S. Second-order many-body perturbation study of ice Ih.

J. Chem. Phys. **137**, 204505 (2012).

[S18] Nanda, K. D. & Beran, G. J. O. Prediction of organic molecular crystal geometries from MP2-level fragment quantum mechanical/molecular mechanical calculations. *J. Chem. Phys.* **137**, 174106 (2012).

[S19] Nanda, K. & Beran, G. What governs the proton-ordering in ice XV. *J. Phys. Chem. Lett.* **4**, 3165–3169 (2013).

[S20] Li, J., Sode, O., Voth, G. A. & Hirata, S. A solid-solid phase transition in carbon dioxide at high pressures and intermediate temperatures. *Nat. Commun.* **4**, 2647 (2013).

[S21] Gilliard, K., Sode, O. & Hirata, S. Second-order many-body perturbation and coupled-cluster singles and doubles study of ice VIII. *J. Chem. Phys.* **140**, 174507 (2014).

[S22] Hirata, S., Gilliard, K., He, X., Li, J. J. & Sode, O. Ab initio molecular crystal structures, spectra, and phase diagrams. *Acc. Chem. Res.* **47**, 2721–2730 (2014).

[S23] Fujita, T., Nakano, T. & Tanaka, S. Fragment molecular orbital calculations under periodic boundary condition. *Chem. Phys. Lett.* **506**, 112–116 (2011).

[S24] Brorsen, K. R., Minezawa, N., Xu, F., Windus, T. L. & Gordon, M. S. Fragment molecular orbital molecular dynamics with the fully analytic energy gradient. *J. Chem. Theory Comput.* **8**, 5008–5012 (2012).

[S25] Brorsen, K. R., Zahariev, F., Nakata, H., Fedorov, D. G. & Gordon, M. S. Analytic gradient for density functional theory based on the fragment molecular orbital method. *J. Chem. Theory Comput.* **10**, 5297–5307 (2014).

[S26] Gao, J. Toward a molecular orbital derived empirical potential for liquid simulations. *J. Phys. Chem. B* **101**, 657–663 (1997).

[S27] Allen, M. P. & Tildesley, D. J. *Computer Simulation of Liquids* (Oxford University Press, New York, 1989).

[S28] Jurečka, P., Špnoer, J., Černý, J. & Hobza, P. Benchmark database of accurate (MP2 and CCSD(T) complete basis set limit) interaction energies of small model complexes, DNA base pairs, and amino acid pairs. *Phys. Chem. Chem. Phys.* **8**, 1985–1993 (2006).

[S29] Fanourgakis, G. S. & Xantheas, S. S. Development of transferable interaction potentials for water. V. Extension of the flexible, polarizable, Thole-type model potential (TTM3-F, v. 3.0) to describe the vibrational spectra of water clusters and liquid water. *J. Chem. Phys.* **128**, 074506 (2008).
